# Influence of day-night and weekday-weekend differences on rapid response team performance in in-hospital cardiac arrest

**DOI:** 10.1590/1806-9282.20250647

**Published:** 2026-03-30

**Authors:** Edson Luiz Fávero, Felipe Antonio Rischini, Thiago Dias Baumgratz, Danilo Martins, Cintia Mitsue Pereira Suzuki, José Martins de Souza, Taline Lazzarin, Paula Schmidt Azevedo, Leonardo Zornoff, Marcos Ferreira Minicucci

**Affiliations:** 1 Universidade Estadual Paulista, Medical School, Department of Internal Medicine – (SP), Brazil.

**Keywords:** Cardiac arrest, Rapid response team, Return of spontaneous circulation, Mortality

## Abstract

**OBJECTIVE:**

The aim of this study was to compare the return of spontaneous circulation and survival to discharge of patients who experienced in-hospital cardiac arrest assisted by a rapid response team during the day versus night and weekdays versus weekends.

**METHODS:**

This retrospective observational study was conducted at a tertiary teaching hospital. Patients aged 18 years or older who experienced a cardiac arrest in the wards between March 2018 and December 2021 were included. Patients with an express order of “natural death permission” were excluded. Data regarding the period of in-hospital cardiac arrest occurrence, return of spontaneous circulation, and survival to discharge were collected from medical records. In addition, data related to nursing staff leave were collected.

**RESULTS:**

A total of 387 patients were included in the analysis. The mean age was 65.4±14.8 years, and 53.7% were men. In the multivariate logistic regression, in-hospital cardiac arrest during the day versus night was not associated with return of spontaneous circulation. However, in-hospital cardiac arrest during weekdays versus weekends showed a significant association with return of spontaneous circulation (odds ratio=2.073; 95%CI 1.206–3.564; p=0.008). No differences were found in survival to discharge between daytime versus nighttime or weekdays versus weekends.

**CONCLUSION:**

There is no difference in return of spontaneous circulation rates between in-hospital cardiac arrest events occurring during the day versus night. However, a significant difference in return of spontaneous circulation rates was observed between weekdays and weekends, although this difference did not extend to in-hospital survival rates.

## INTRODUCTION

In-hospital cardiac arrest (IHCA) is an acute event that can potentially affect any hospitalized patient^1^. It is defined in the Utstein resuscitation registry reporting template as the delivery of chest compressions and/or defibrillation to patients admitted to inpatient beds^2^. IHCA is associated with a high risk of death. However, mortality rates have decreased due to increased awareness of the influence of clinical management on patient outcomes^3^. Recent data suggest improvements over the past two decades^1^.

Despite these improvements, hospital survival is lower for patients admitted during weekends and at night^4^. Data from the UK National Cardiac Arrest Audit indicate that crude hospital survival is worse after IHCA at night versus daytime and on weekends versus weekdays, despite a similar frequency of events^5^.

The extent to which differences in care between day and night or between weekdays and weekends result in variations in return of spontaneous circulation (ROSC) and survival among IHCA patients remains debated. At night, hospital staffing patterns differ, with fewer admissions, discharges, and diagnostic and therapeutic procedures compared with other times^6^. Staffing levels in acute care hospitals also tend to be lower on weekends than on weekdays, and the reduction in clinical personnel may lead to shortfalls in care^7^.

The implementation of rapid response teams (RRTs) has been reported as a key factor in improving resuscitation performance. Dedicated teams have been identified as a feature of centers with higher IHCA survival^8^. However, even with an RRT, there is limited evidence regarding resuscitation performance according to the period of the day or the day of the week. Recent studies have shown conflicting results regarding RRT performance^4,9^.

In this study, all IHCAs were managed by the RRT, regardless of the time of occurrence. We hypothesized that, with the same intervention team, the outcomes of IHCA would not differ according to the period of occurrence. Thus, the objective of this study was to compare ROSC and survival to discharge of patients with IHCA managed by the RRT during the day versus night and weekdays versus weekends.

## METHODS

### Study design and population

This retrospective observational study was conducted at a tertiary teaching hospital. It was initiated after approval by the Ethics Committee of our institution (protocol code: 56979721.90000.5411). The Ethics Committee authorized a waiver of the written consent form.

We included all patients aged 18 years or older with a record of “rapid response team—code blue” in the electronic medical record system MV^®^ from March 2018 to December 2021. This record is entered by RRT staff for all patients who experienced an IHCA managed by the RRT in the wards. Only the index pulseless events were included. An index event was defined as the first cardiac arrest for patients with more than one arrest during the same hospitalization. Exclusion criteria included all arrests of patients in palliative care with an express order of “natural death permission” in the electronic medical record.

### Variables and outcomes

The record generated a spreadsheet that could be exported to Excel containing data on identification, age, sex, rhythm of arrest, ward, resuscitation time, defibrillation events, medications and doses used, indication for intensive care, and advanced airway strategy. Comorbidities, laboratory test results, and length of stay were also collected.

Patients were categorized according to outcomes. The main outcomes were ROSC and in-hospital mortality. ROSC was defined as the restoration of a pulse lasting more than 20 min. The day and time of IHCA were categorized as weekday (Monday to Friday), weekend (Saturday and Sunday), daytime (07:00– 19:00), and nighttime (19:00–07:00). These periods were defined based on the shift changes of medical and nursing teams.

In addition, we collected data from the Specialized Work Safety Center (SESMT) of the HCFMB (Hospital das Clínicas da Faculdade de Medicina de Botucatu), which provided information on nursing staff absences for the years 2019, 2020, and 2021.

### Statistical analysis

All statistical analyses were performed using SigmaPlot software for Windows v12.0 (Systat Software Inc., San Jose, CA, USA). Categorical data were expressed as frequency (%). Continuous data with a normal distribution were expressed as mean and standard deviation, whereas continuous variables with a non-normal distribution were expressed as median and interquartile range (25th and 75th percentiles). Data normality was assessed using the Shapiro-Wilk test.

For comparisons between groups, the unpaired Student’s t-test or Mann-Whitney U test was applied when variables were continuous, and the chi-square test or Fisher’s exact test when variables were categorical. For comparisons between the proposed study periods (day versus night and weekday versus weekend) and IHCA outcomes (ROSC and survival), multiple logistic regression was performed. Two models were developed for each exposure and outcome. The first model was adjusted for age, sex, duration of IHCA, and rhythm, as these are relevant prognostic variables in cardiac arrest. The second model included variables with statistical differences in univariate analyses (Student’s t-test, Mann-Whitney U test, chi-square test, or Fisher’s exact test) for each outcome.

The magnitude of the effect of each variable was estimated by calculating the odds ratio (OR) with the respective 95%CI. The significance level adopted was 5%.

## RESULTS

**Figure 1:**
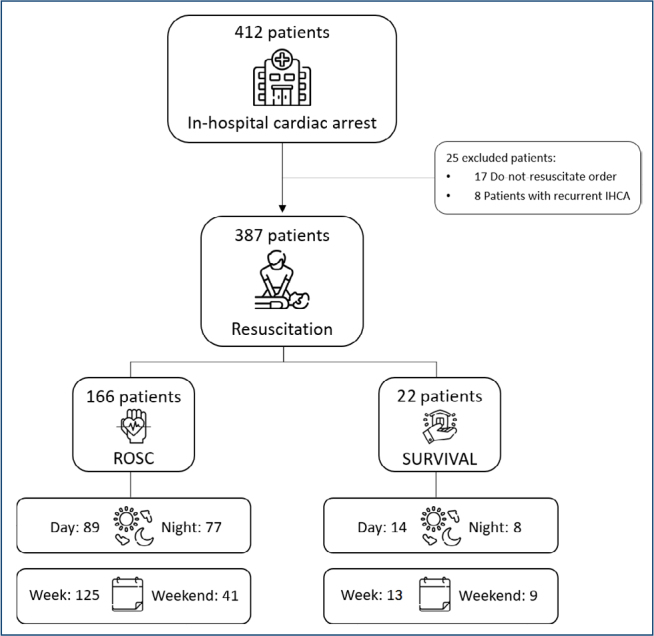
Flowchart of patients treated at in-hospital cardiac arrest.

A total of 412 patients with IHCA assisted by the RRT were evaluated. Of these, 25 patients were excluded: 17 due to “order to allow natural death” and 8 because the event was not the index episode (Figure 1). Thus, 387 patients were included in the analysis. The mean age was 65.4±14.8 years, 53.7% were men, 91.2% presented non-shockable rhythms, 50.6% of the events occurred during the day, and 70.5% occurred on weekdays. Among the outcomes evaluated, the ROSC rate was 42.8%, and hospital survival was 5.7%.

According to Table 1, younger patients showed higher ROSC rates. Statistical significance was also observed among patients resuscitated for a shorter time. Regarding survival to discharge, as described in Table 2, mortality was higher among older patients, those with longer IHCA duration, those with hypertensive disease, and those who received adrenaline during resuscitation. Survival was higher among patients with lower urea, creatinine, and prothrombin time values.

**Table 1:** Demographic and clinical data of 387 patients between March 2018 and December 2021 with in-hospital cardiac arrest according to return of spontaneous circulation.

Variables	ROSC		p-value
	Yes (166)	No (221)	
Age, years	65 (55–73)	69 (58–78)	**0.007**
Male, n (%)	86 (51.8%)	122 (55.2%)	0.575
Duration of CPR, min	14 (7–21)	30 (20–35)	**0.001**
Hypertension, n (%)	101 (60.8%)	145 (65.6%)	0.391
Diabetes, n (%)	56 (33.7%)	140 (63.3%)	0.627
Smoking, n (%)	59 (35.5%)	71 (32.1%)	0.552
Time of day			
Day, n (%)	89 (53.6%)	107 (48.5%) 114	0.363
Night, n (%)	77 (46.4%)	(51.5%)	
Time of week			
Weekday, n (%)	125 (75.4%)	146 (66.1%)	0.064
Weekend, n (%)	41 (24.6%)	75 (33.9%)	
Illness category			
Medical	82 (49.4%)	105 (47.5%)	0.663
Surgical	84 (50.6%)	116 (52.5%)	
Rhythm			
Non-shockable	148 (89.1%)	205 (92.7%)	0.290
Shockable	18 (10.9%)	16 (7.3%)	
Adrenaline, n (%)	162 (97.59%)	220 (99.5%)	0.218
Hemoglobin, mg/dL	11.96±2.5	11.8±2.5	0.605
Hematocrit, %	36.48±7.42	36.23±7.39	0.751
Urea, mg/dL	54 (35–95)	57.5 (35.7–96.7)	0.240
Creatinin, mg/dL	1 (0.7–1.7)	1.1 (0.7–2.2)	0.437
Sodium, mmol/L	136 (131–139)	136 (133–139)	0.139
Potassium, mmol/L	4.3 (3.9–4.90)	4.4 (3.9–4.95)	0.643
PT	1.34 (1.14–1.89)	1.26 (1.145–1.615)	0.475
APTT	1.005 (0.93–1.138)	0.995 (0.92–1.12)	0.502

CPR: cardiopulmonary resuscitation; ROSC: return of spontaneous circulation; PT: prothrombin time; APTT: total activated thromboplastin time. Data are expressed as mean±SD, median (25–75%), and percentage. Bold in p-value column represents p-values with statistical significance (<0.05).

**Table 2:** Demographic and clinical data of the 387 patients between March 2018 and December 2021 with in-hospital cardiac arrest according to survival to discharge.

Variables	Survival to discharge		p-value
	Yes (22)	No (365)	
Age, years	53 (43.5–68.5)	67.0 (57–77)	**<0.001**
Male, n (%)	10 (45.5%)	198 (54.2%)	0.560
Duration of CPR, min	6 (2–12.5)	24 (15–32)	**<0.001**
Hypertension, n (%)	8 (36.4%)	238 (65.2%)	**0.012**
Diabetes, n (%)	5 (22.7%)	132 (36.2%)	0.294
Smoking, n (%)	11 (50%)	119 (32.6%)	0.148
Time of day			
Day, n (%)	14 (63.6%)	182 (49.9%)	0.301
Night, n (%)	8 (36.4%)	183 (50.1%)	
Time of week			
Weekday, n (%)	13 (59.1%)	258 (70.7%)	0.361
Weekend, n (%)	9 (40.9%)	107 (29.3%)	
Illness category			0.637
Medical	9 (40.9%)	177 (48.5%)	
Surgical	13 (59.1%)	188 (51.5%)	
Rhythm			0.737
Non-shockable	20 (90.9%)	333 (91.2%)	
Shockable	2 (9.1%)	32 (8.8%)	
Adrenaline, n (%)	20 (90.9%)	362 (99.2%)	**0.018**
Hemoglobin, mg/dL	12.2±1.91	11.9±2.6	0.580
Hematocrit, %	36.9±5.2	36.3±7.5	0.713
Urea, mg/dL	27.5 (22.3–51.3)	57.0 (38.0–96.8)	**<0.001**
Creatinin, mg/dL	0.7 (0.6–1.3)	1.1 (0.8–1.9)	**0.005**
Sodium, mmol/L	136 (131–137)	136 (133–139)	0.136
Potássium, mmol/L	4.3 (4.0–4.9)	4.3 (3.9–4.9)	0.947
PT	1.04 (0.99–1.09)	1.16 (1.05–1.36)	**0.003**
APTT	0.98 (0.89–1.01)	1.01 (0.92–1.13)	0.157

CPR: cardiopulmonary resuscitation; PT: prothrombin time; APTT: total activated thromboplastin time. Data are expressed as mean±SD, median (25–75%), and percentage.

Bold in p-value column represents p-values with statistical significance (<0.05).

Two multivariate logistic regression models were applied for each outcome. In the second model for ROSC, the variables included were age and duration of IHCA. In the second model for mortality, the variables were age, hypertension, duration of IHCA, use of adrenaline, urea, and prothrombin time. Creatinine was excluded due to collinearity with urea.

For ROSC, no significant difference was found when comparing day versus night, even after adjustment for confounders (Table 3). However, there was a significant association between ROSC and weekday occurrence of IHCA. In both regression models, there was at least a two-fold increase in the likelihood of ROSC when IHCA occurred during a weekday (Table 3). For survival to discharge, using both multivariate logistic regression models, no significant differences were observed when comparing day versus night or weekdays versus weekends (Table 3).

**Table 3:** Multivariate logistic regression for return of spontaneous circulation and in-hospital survival to discharge.

	ROSC		
	OR	95%CI	p-value
Day^*^	1.111	0.681–1.811	0.674
Day^**^	1.124	0.691–1.830	0.637
Weekday^*^	2.067	1.200–3.560	**0.009**
Weekday^**^	2.073	1.206–3.564	**0.008**
	**Survival to discharge**		
	**OR**	**95%CI**	**p-value**
Day^*^	1.760	0.721–4.296	0.215
Day^***^	0.670	0.145–3.086	0.607
Weekday^*^	0.599	0.249–1.443	0.253
Weekday^***^	0.476	0.098–2.316	0.358

*Adjusted by age, gender, duration of in-hospital cardiac arrest, and rhythm.

**Adjusted by age and duration of in-hospital cardiac arrest.

***Adjusted by age, duration of in-hospital cardiac arrest, hypertension, adrenaline, urea, and prothrombin time. ROSC: return of spontaneous circulation; OR: odds ratio; CI: confidence interval. Bold in p-value column represents p-values with statistical significance (<0.05).

Data obtained from SESMT showed a higher rate of hours of leave among nursing professionals due to illness on weekends compared to weekdays.

## DISCUSSION

The objective of this study was to evaluate the rates of ROSC and survival in patients with IHCA assisted by an RRT, comparing day versus night and weekdays versus weekends. Our results showed no statistically significant difference between day and night. However, a significant difference was observed when comparing weekday versus weekend ROSC, although survival to discharge did not differ.

Every year, more than 200,000 IHCAs are registered in the United States alone, and these numbers continue to increase^10^. Survival rates are directly related to early recognition, high-performance chest compressions, and specialized post-resuscitation care^11^. Despite advances in resuscitation techniques, survival is 15–20% lower among patients experiencing IHCA at night and on weekends^10,12^. Understanding the factors contributing to these differences can substantially improve the safety of hospital services.

Wards do not routinely have ICU monitoring technologies. In the absence of monitors, healthcare professionals must adapt their usual functions to monitor patients’ condition, as reported by Robinson et al.^4^. A reduced number of nursing staff working at night and on weekends can delay the recognition of IHCA, the initiation of resuscitation maneuvers, and RRT activation. Determining the ideal patient-to-nurse ratio in wards could positively impact care indicators. This relationship remains unclear in the literature and requires further investigation^13,14^.

In our study, no statistically significant differences were observed between cardiac arrests occurring during the day versus night. Both ROSC and survival outcomes were similar across these groups. This finding differs from the conclusions of Peberdy et al., who observed differences for the same outcomes. However, in a subgroup analysis of that study, the authors reported no differences between day and night for IHCAs occurring in the Emergency Department and Trauma Services, attributing the results to the high performance and constant availability of teams in these sectors^14^. A similar positive outcome was observed with the RRT in our study.

The prompt response of a highly trained team enhances care. The RRT approach is associated with improved IHCA outcomes in high-performing hospitals^15^. Studies have shown that the implementation of RRTs can lead to a significant reduction in the incidence of IHCA and overall hospital mortality^16,17^.

In our study, the rates of IHCA with non-shockable rhythms were very high compared with previous research, which reports rates ranging from 60 to 80%. Our sample identified non-shockable rhythms in approximately 90% of IHCAs. This finding may reflect the severity of illness among our inpatients. Patients who develop these rhythms have a lower chance of ROSC, which likely contributed to the mortality rates exceeding 90%. Nevertheless, the RRT ensured ROSC rates greater than 43%, comparable to resuscitation outcomes reported in intensive care units^18^.

Our study also included patients with COVID-19. The ROSC and mortality outcomes of these patients were similar to those reported in the literature^19^. A sensitivity analysis excluding these patients was performed to evaluate their potential influence on the study objectives. In our sample, COVID-19 did not alter the relationship between IHCA occurrence and the outcomes ROSC and survival to discharge (data not shown).

ROSC rates were lower during weekends. Even with the support of the RRT, this outcome was worse when IHCAs occurred on Saturdays or Sundays. We also observed a higher rate of nursing staff leaving due to illness on weekends compared with weekdays. When starting a workday and being informed of unforeseen leave, staff are often not replaced promptly. Despite efforts, this staffing gap is not corrected in time, and the deficit directly affects the quality of care. We hypothesize that fewer professionals in the wards could delay the recognition of clinical deterioration and, consequently, the activation of the RRT. Further studies are needed to elucidate how the nurse-to-patient ratio in wards influences ROSC.

It is important to emphasize the limitations of this study. This was a retrospective observational study including patients from a single tertiary hospital with a relatively small sample. Additionally, the single-center setting and relatively small sample size may introduce selection bias. Therefore, these results may not reflect the reality of other centers. Finally, the extremely high mortality could reduce the statistical power of the study.

## CONCLUSION

No significant difference in ROSC rates was observed between IHCA events assisted by the RRT in our hospital during the day versus night. However, higher ROSC rates were associated with IHCA events occurring on weekdays compared with weekends, although this association did not translate into differences in in-hospital survival rates.

## DECLARATION OF GENERATIVE AI AND AI-ASSISTED TECHNOLOGIES IN THE WRITING PROCESS

During the preparation of this work, the authors used OpenAI ChatGPT 5.0 to review and improve the language. After using this tool, the authors reviewed and edited the content as needed and take full responsibility for the content of the published article.

## Data Availability

The datasets generated and/or analyzed during the current study are available from the corresponding author upon reasonable request.
